# 固相萃取-离子迁移谱法快速筛查祛痘类化妆品中14种抗菌药物

**DOI:** 10.3724/SP.J.1123.2022.05025

**Published:** 2022-12-08

**Authors:** Gaoxu XUE, Qinyi WANG, Ling CAO, Jing SUN, Gongjun YANG, Youlong FENG, Fang FANG

**Affiliations:** 1.江苏省食品药品监督检验研究院, 江苏 南京 210019; 1. Jiangsu Institute for Food and Drug Control, Nanjing 210019, China; 2.南京中医药大学附属医院/江苏省中医院, 江苏 南京 210029; 2. Affiliated Hospital of Nanjing University of Chinese Medicine, Jiangsu Province Hospital of Chinese Medicine, Nanjing 210029, China; 3.中国药科大学药学院, 江苏 南京 211198; 3. School of Pharmacy, China Pharmaceutical University, Nanjing 211198, China

**Keywords:** 固相萃取, 离子迁移谱, 抗菌药物, 祛痘化妆品, 快速筛查, solid-phase extraction (SPE), ion mobility spectrometry (IMS), antibacterial drugs, anti-acne cosmetics, rapid screening

## Abstract

目前,主动性的现场稽查已成为市场监管的发展趋势,这需要在现场快速有效地筛查大量产品,评估是否含有非法添加化学药物,对有嫌疑的样品及时封存,再送至实验室进一步检验。离子迁移谱技术是近年来发展起来的快筛技术之一。实验采用固相萃取-离子迁移谱技术,建立了祛痘类化妆品中14种抗菌药物的快速筛查方法。对离子迁移谱检测条件、样品提取条件、固相萃取净化条件(固相萃取柱、淋洗液种类、洗脱液种类及体积)进行了详细考察与优化。最终使用80%(体积分数)乙腈水溶液(含0.2%(质量分数)三氯乙酸)作为样品提取溶液,提取后上样于活化后的弱阳离子交换柱(Oasis^®^ MCX固相萃取柱), 3.0 mL甲醇淋洗,1.0 mL 2%氨水甲醇洗脱,洗脱液直接进离子迁移谱检测。14种抗菌药物的迁移时间在11~17 ms之间,检出限为0.2~1.2 μg/g。同时,由于离子迁移谱法线性范围较窄,不能准确定量,建立了高效液色谱(HPLC)定量方法,用于固相萃取前处理步骤的优化和阳性样品的验证。25批化妆品样品中,筛查出1批阳性样品,与HPLC检测结果相符。该方法快速、简便、高效,显著降低了祛痘类化妆品基质对离子迁移谱检测14种抗菌药物的干扰,提高了检测灵敏度,有效降低了假阳性和假阴性的发生,可用于化妆品现场快速筛查,同时也扩大了离子迁移谱在化妆品等复杂基质中非法添加化学药物检测的应用范围。

## Abstract (en)

The main methods currently used to detect illegally added chemicals in cosmetics include thin-layer chromatography, high performance liquid chromatography (HPLC), gas chromatography (GC), and liquid chromatography-mass spectrometry (LC-MS). Compared with other analytical techniques, these methods have the advantages of high sensitivity, specificity, and accuracy, all of which are required in practical detection work. However, they also present a number of limitations, such as long analysis times and requirements for skilled operators and strictly controlled laboratory environments. Supervision, a growing trend in market surveillance, requires rapid and effective methods to screen illegally added chemicals. The suspected samples are sealed for some time and then sent to the laboratory for further testing. Ion mobility spectrometry (IMS) is a new type of trace gas separation technology that was developed in recent years. The principle behind IMS is the separation and characterization of chemical species based on differences in the migration speed of their gas-phase ions under an electric field. As this technology has the advantages of miniaturization, easy operation, and quick responses, it is widely used in food and drug quality testing, as well as other related fields. However, it is rarely used in cosmetic detection, likely because the cosmetics matrix is highly complex, which can interfere with ion determination. Thus, optimizing the pretreatment process of cosmetics for IMS is important. In this work, solid-phase extraction (SPE) is combined with IMS to establish a method for the rapid screening of 14 antibacterial drugs in anti-acne cosmetics. The IMS detection parameters, sample extraction conditions, and SPE clean-up conditions (SPE column, type of leachate, type and volume of eluent) were studied and optimized in detail. The sample was extracted with 80%(v/v) acetonitrile aqueous solution (containing 0.2% (mass fraction) trichloroacetic acid), loaded onto an activated Oasis^®^ MCX SPE column, leached with 3.0 mL of methanol, and eluted with 1.0 mL of 2% ammonia methanol solution. The eluate was then directly injected into the IMS instrument. The IMS parameters were as follows: positive ion source voltage=2200 V, transfer tube voltage=8000 V, inlet temperature=180 ℃, transfer tube temperature=180 ℃, ion gate voltage=50 V, gate voltage pulse width=85 μs, and migration gas flow rate=1.2 L/min. The migration times for the 14 antibacterial drugs ranged from 11 to 17 ms, and the detection limits for the target compounds ranged from 0.2 to 1.2 μg/g. Owing to the narrow linear range of IMS, a quantitative method employing HPLC was also established to optimize the SPE pretreatment step and verify the positive samples. Chromatographic separation was conducted on a Phenomenex Luna C_18_ column (250 mm×4.6 mm, 5 μm), with a column flow rate of 1.0 mL/min and gradient elution with mobile phases A (0.01 mol/L potassium dihydrogen phosphate adjusted to pH 4.0 with phosphoric acid) and B (acetonitrile). The column temperature was set to 35 ℃, and the injection volume was fixed at 5 μL. A total of 25 cosmetics samples were screened, and one positive sample was found to be consistent with the results of HPLC. The proposed method is fast, simple, and efficient, and it can be used for the rapid screening of the 14 antibacterial drugs in anti-acne cosmetics. Pretreatment can significantly reduce the influence of the cosmetic matrices on the determination results, improve instrument sensitivity, and effectively decrease the occurrence rate of false positives and negatives. The technique developed in this work can improve the efficiency of screening for illegally added chemicals and expand the applications of IMS for detecting various chemicals in complex matrices, such as cosmetics.

随着国民经济的迅猛发展,人们对于美的追求越来越高,化妆品需求量急剧增加,一些不法商家为了迎合消费者的迫切需求,在化妆品中非法添加各种违禁的化学药物,以暂时性提高化妆品功效,导致消费者使用后出现一些不良反应^[1]^。其中,禁用成分如抗真菌、抗生素类等抗菌药如果随意添加到祛痘类产品中,容易引起接触性皮炎,表现为红斑、水肿、糜烂、脱屑、瘙痒、灼热等症状^[2]^,甚至造成更严重的系统健康问题。现阶段用于检测化妆品中非法添加化学药物的技术主要为色谱技术和色谱-质谱联用技术^[3⇓⇓-6]^。这些方法检测时间长,仪器操作难度高,受场地等因素限制,不适用于现场快速筛查。现阶段实际投入非法添加现场筛查的技术较少,主要以理化鉴别技术为主^[7]^,即利用待测化学成分的溶解度、官能团等性质与基质成分的不同,通过适当的样品前处理技术排除基质干扰,再根据待测化学成分的理化性质进行鉴别的技术。这些方法操作简单,研发和检测成本低,试剂盒种类多;但是检出限较低,通用性较差,在有颜色的基质下受到干扰严重,在化妆品检测中应用较少。因此,亟需新技术的引进。

离子迁移谱(ion mobility spectrometry, IMS)是20世纪中期发展起来的一种新型的微量气相分离分析技术,因其具有小型化、操作简便、响应迅速等优点,可作为快筛方法应用于公共安全^[8]^等检测。IMS仪是对气相离子进行分析检测的装置,气相组分可直接进入迁移管进行离子化并检测,而对于液体及固体样品则可分别经电喷雾离子化(electrospray ionization, ESI)和基质辅助激光解吸离子化等技术实现样品导入。ESI作为一种软电离源,能够获得被分析物的整体分子信息,非常适用于非挥发性或低挥发性化合物的分析^[9]^,如常见化学药物。已有相关文献报道ESI-IMS成功应用于中药^[10]^、化学药品检测^[11⇓-13]^、食品及化妆品安全^[14⇓⇓⇓-18]^等相关领域。但是目前ESI-IMS直接应用于化妆品中的禁用化学药物检测的成功案例较少。

推测阻碍ESI-IMS应用于化妆品中违禁物质筛查的原因主要有以下几点:(1)ESI-IMS存在明显的记忆效应^[19]^(主要为离子抑制效应),化妆品经溶剂提取直接液体进样,其基质会严重污染离子源,影响仪器的灵敏度和检测结果的重现性;(2)化妆品基质常存在大量的表面活性剂,会产生一系列离子迁移谱峰,干扰违禁目标物质的判定,造成假阳性结果;(3)化妆品中添加的禁用物质浓度较低,样品基质会严重抑制目标化合物在离子源中电离,单纯提高样品取样量无法提高检测灵敏度,易造成假阴性结果。因此很有必要优化样品的净化过程,以适用于现场的快速筛查。化妆品基质复杂,常采用固相萃取(SPE)的方式进行净化^[20]^。

根据日常检验工作及文献报道,非法添加抗菌药物的添加量一般在μg/g甚至mg/g量级^[21]^。本文建立了SPE结合IMS应用于祛痘类化妆品中14种抗菌药物的快速筛查方法。由于离子迁移谱线性范围较窄^[22]^,不能准确定量,同时建立了高效液相色谱法(HPLC)的定量方法,用于SPE前处理的优化和阳性样品的验证。方法简便、快速、灵敏,提高了非法添加化学药物筛查工作的效率,为监管部门打击化妆品生产经营中添加违禁化学药物的违法行为提供了科学有效的技术手段。

## 1 实验部分

### 1.1 仪器、试剂与材料

GA2100高效离子迁移谱仪配有电喷雾离子源、Vision仪器控制与数据处理系统(美国Excellims公司);AG-1620型空气发生器(北京科普生分析科技有限公司);LC-30A高效液相色谱仪(日本Shimadzu公司); XP6型电子天平(瑞士Mettler Toledo公司); MSE224S-CE电子天平(德国Sartorius公司);超纯水系统(美国Millipore公司); UA22MFD超声波清洗仪(德国Wiggens公司),固相萃取仪(美国Supelco公司)。Oasis^®^ MCX固相萃取柱(3 mL/60 mg, 30 μm)购自美国Waters公司。

盐酸双氟沙星(difloxacin hydrochloride,批号:G752673)和盐酸沙拉沙星(sarafloxacin hydrochloride,批号:G752046)来源于Dr. Ehrenstorfer公司;氧氟沙星(ofloxacin,批号:130454-202007)、诺氟沙星(norfloxacin,批号:130450-201907)、培氟沙星(pefloxacin,批号:130459-201402)、依诺沙星(enoxacin,批号:130453-201802)、环丙沙星(ciprofloxacin,批号:130451-201904)、氟康唑(fluconazole,批号:100314-201906)、盐酸萘替芬(naftifine hydrochloride,批号:100823-200501)、酮康唑(ketoconazole,批号:100294-201904)、联苯苄唑(bifonazole,批号:100326-201302)、克霉唑(clotrimazole,批号:100037-202008)、硝酸益康唑(econazole nitrate,批号:100214-202005)、硝酸咪康唑(miconazole nitrate,批号:100213-201406)、色氨酸(tryptophan,批号:140686-201904)均来源于中国食品药品检定研究院。甲醇(Merck公司,德国)、乙腈(Merck公司,德国)、氨水(阿拉丁试剂有限公司)均为色谱纯。磷酸二氢钾(国药集团化学试剂有限公司)、磷酸(阿拉丁试剂有限公司)、三氯乙酸(阿拉丁试剂有限公司)均为分析纯。

25批祛痘类化妆品均为市售,化妆品类型包含乳剂、水剂和膏霜剂,其中乳剂8批、水剂8批、膏霜剂9批。

### 1.2 标准溶液的配制

#### 1.2.1 IMS标准溶液

称取各标准品约10.0 mg,分别置于25 mL量瓶中,用80%(v/v,下同)甲醇水溶液溶解并稀释至刻度,配制成400 mg/L的标准储备液(沙星类物质可加少量甲酸助溶)。分别量取0.50、1.00、2.00 mL至20 mL量瓶中,用80%甲醇水溶液稀释至刻度,摇匀,作为各物质的IMS标准溶液。

#### 1.2.2 IMS校正溶液

称取色氨酸约2.0 mg,分别置于10 mL量瓶中,用80%甲醇水溶液溶解并稀释至刻度,配制成200 mg/L的IMS正离子模式校正溶液。

#### 1.2.3 HPLC标准溶液

称取各标准品约1.0 mg,分别置于10 mL量瓶中,用甲醇稀释至刻度,配制成100 mg/L的单标准溶液(沙星类物质可加少量甲酸助溶),另称取依诺沙星、诺氟沙星、环丙沙星、氟康唑、酮康唑、联苯苄唑、硝酸益康唑、硝酸咪康唑标准品各5.0 mg,置于50 mL量瓶中,用甲醇稀释至刻度,配制成100 mg/L的混合标准储备液Ⅰ(沙星类物质可加少量甲酸助溶),然后用甲醇逐级稀释至质量浓度约为5.0~80.0 mg/L的系列混合标准工作溶液Ⅰ。称取氧氟沙星、培氟沙星、盐酸双氟沙星、盐酸沙拉沙星、盐酸萘替芬、克霉唑标准品各5.0 mg,分别置于50 mL量瓶中,用甲醇稀释至刻度,配制成100 mg/L的混合标准储备液Ⅱ,然后用甲醇逐级稀释,配制质量浓度约为5.0~80.0 mg/L的系列混合标准工作溶液Ⅱ。

### 1.3 样品前处理

取0.25 g样品于15 mL离心管中,加入5.0 mL 80%(体积分数,下同)乙腈水溶液(含0.2%(质量分数)三氯乙酸),涡旋,混匀,超声10 min后,5000 r/min离心10 min,上清液待SPE柱净化。

Oasis^®^MCX柱依次用3.0 mL甲醇、3.0 mL 1%(质量分数,下同)三氯乙酸水溶液活化。将待净化的样品上清液流经SPE柱后,用3.0 mL甲醇淋洗SPE柱,弃去淋洗液,再用1.0 mL 2%氨水甲醇洗脱,收集洗脱液,摇匀,待测。

### 1.4 分析条件

#### 1.4.1 离子迁移谱条件

离子源为正离子模式,源电压为2.2 kV,迁移管电压为8.0 kV,进气口温度为180 ℃,迁移管温度为180 ℃,离子栅门电压为50 V,门电压脉冲宽度为85 μs,迁移气流速为1.2 L/min,排出气流速为0.8 L/min,进样速度为1.2 μL/min。仪器使用前用IMS校正溶液在正离子模式下校正仪器。

#### 1.4.2 HPLC验证条件

色谱柱为Phenomenex luna C_18_柱(250 mm×4.6 mm, 5 μm);流动相A: 0.01 mol/L磷酸二氢钾(磷酸调节pH值为4.0),流动相B:乙腈;梯度洗脱:0~3 min, 20%B; 3~18 min, 20%B~95%B; 18~21 min, 95%B; 21~22 min, 95%B~20%B; 22~30 min, 20%B;流速为1.0 mL/min;进样量为5 μL;柱温为35 ℃;其中氟康唑的检测波长为210 nm,其余化合物为230 nm。

## 2 结果与讨论

### 2.1 实验条件考察

#### 2.1.1 IMS检测模式选择

分别于正离子模式与负离子模式下检测各抗菌药物的出峰情况以及目标物的峰形。实验结果发现14种抗菌药物在正离子模式下均有较好的响应,因此,在正离子模式下检测14种抗菌药物。

#### 2.1.2 IMS离子源电压优化

在正离子模式下比较了离子源电压(1.6 ~3.0 kV)对各抗菌药物响应的影响,实验结果如图1所示。结果显示,除克霉唑在2.6 kV时响应最好外,其他抗菌药物在离子源电压为2.2 kV时响应强度较好,综合考虑,选择2.2 kV作为离子源电压。

**图1:**
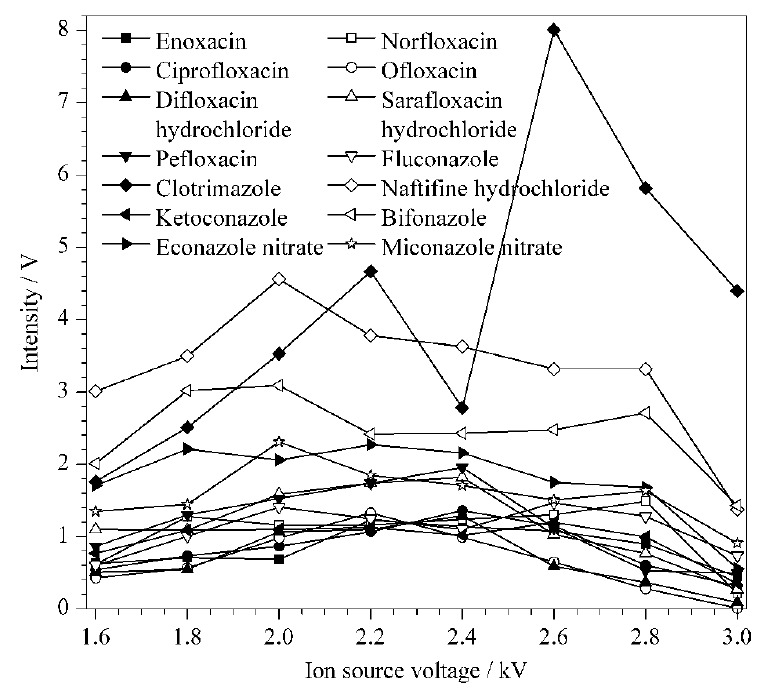
不同离子源电压下14种抗菌药物的ESI-IMS响应

#### 2.1.3 HPLC验证条件优化

实验对流动相进行了优化,比较了不同pH值的0.01 mol/L磷酸二氢钾溶液,结果发现在pH值为4.0时各物质分离较好,但沙星类物质仍未能完全分开,因此将14种抗菌药物分成两组进行分离,依诺沙星、诺氟沙星、环丙沙星、氟康唑、酮康唑、联苯苄唑、硝酸益康唑、硝酸咪康唑为一组,氧氟沙星、培氟沙星、盐酸双氟沙星、盐酸沙拉沙星、盐酸萘替芬、克霉唑为另一组。各物质的回归方程、线性范围、检出限见表1。

**表1:** HPLC测定14种抗菌药物的线性方程、线性范围、检出限

No.	Compound	Linear equation	r^2^	Linear range/(mg/L)	LOD/(mg/L)
1	fluconazole	y=8.23×10^4^x-1.36×10^4^	0.9997	5.068-81.09	0.30
2	norfloxacin	y=1.28×10^5^x-3.71×10^4^	0.9997	5.092-81.46	0.20
3	ciprofloxacin	y=9.66×10^4^x-4.07×10^4^	0.9998	4.208-67.32	0.25
4	pefloxacin	y=7.08×10^4^x-9.43×10^4^	0.9993	3.609-57.75	0.50
5	bifonazole	y=8.61×10^4^x+4.64×10^4^	0.9995	4.998-79.96	0.10
6	sarafloxacin hydrochloride	y=1.00×10^5^x-6.43×10^4^	0.9994	4.340-79.35	0.50
7	miconazole nitrate	y=1.56×10^5^x+5.20×10^4^	0.9996	5.078-81.25	0.20
8	clotrimazole	y=7.34×10^4^x-1.11×10^4^	0.9996	5.059-80.93	0.15
9	enoxacin	y=5.33×10^4^x-3.24×10^4^	0.9998	4.631-74.10	0.40
10	naftifine hydrochloride	y=3.71×10^5^x-9.53×10^2^	0.9991	5.094-81.49	0.03
11	econazole nitrate	y=1.20×10^5^x-7.75×10^2^	0.9997	5.077-81.23	0.20
12	ofloxacin	y=1.95×10^5^x-1.69×10^5^	0.9995	5.078-81.25	0.13
13	difloxacin hydrochloride	y=9.87×10^4^x-7.37×10^4^	0.9995	4.960-79.35	0.25
14	ketoconazole	y=1.04×10^5^x-4.82×10^2^	0.9993	5.072-81.16	0.15

*y*: peak area; *x*: mass concentration, mg/L; *r*^2^: correlation coefficient.

#### 2.1.4 固相萃取柱的选择

考察了Oasis^®^HLB (3 mL/60 mg)、Oasis^®^MCX (3 mL/60 mg)、Oasis^®^WCX (3 mL/60 mg)、Cleanert^®^ ODS (3 mL/300 mg) SPE柱对化妆品中基质的净化效果。取0.25 g阴性样品分别经Oasis^®^HLB、Oasis^®^WCX、Oasis^®^MCX、Cleanert^®^ ODS SPE柱处理,在1.4.1节条件下进行IMS分析,实验结果如图2所示,发现Oasis^®^HLB、Oasis^® ^WCX SPE柱都无法去除化妆品中的基质干扰,Cleanert^®^ ODS和Oasis^®^MCX SPE两柱可以去除化妆品中的基质干扰,考虑到待测物的性质,实验选择Oasis^®^MCX SPE柱做进一步优化。

**图2:**
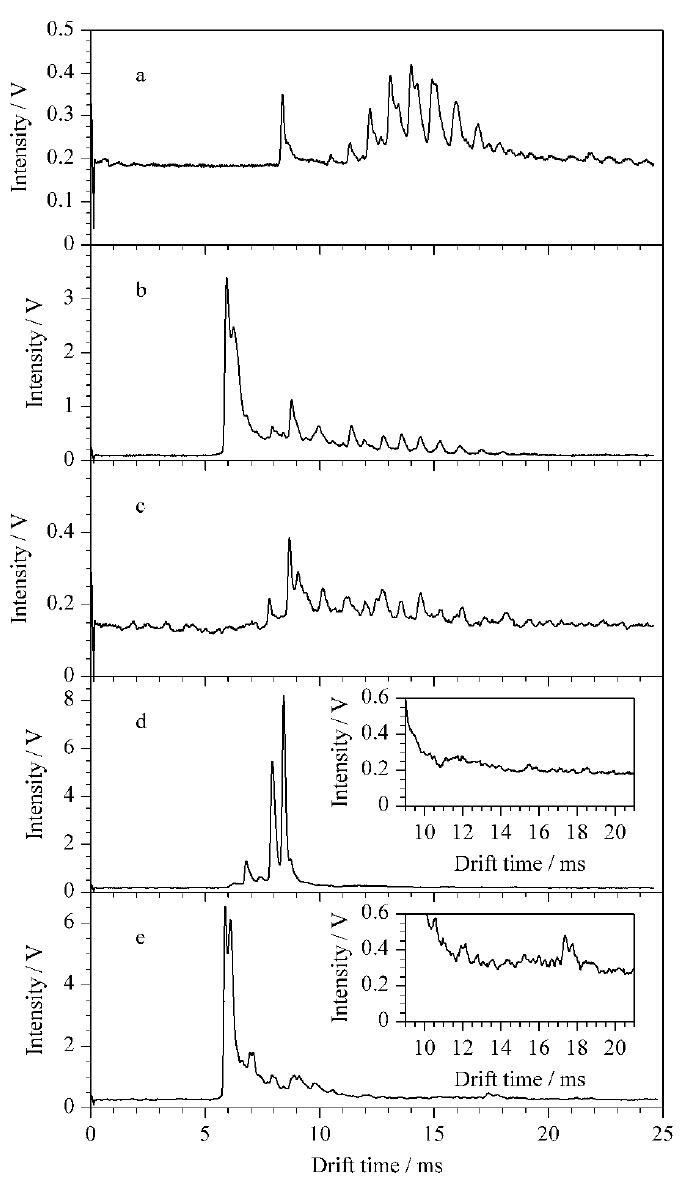
阴性样品经(a)直接提取、(b)HLB柱、(c)ODS柱、(d)MCX柱和(e)WCX柱萃取的IMS谱图

进一步考察了不同规格的Oasis^®^MCX SPE柱(3 mL/60 mg, 30 μm)与Oasis^®^MCX SPE柱(6 mL/150 mg, 30 μm)对回收率的影响。分别取200.0 μL混合标准储备液Ⅰ与混合标准储备液Ⅱ于阴性样品中,按1.3节方法处理,在1.4.2节条件下进行HPLC分析。结果发现,规格为3 mL/60 mg的MCX固相萃取柱所需的洗脱溶液体积更小,实验灵敏度更高。因此,实验选择SPE柱的规格为3 mL/60 mg。

#### 2.1.5 SPE提取条件的优化

甲醇和乙腈是化妆品常用的提取溶剂,在实验操作中发现,甲醇提取的溶液经离心后有浑浊现象,因此选乙腈作为样品提取溶剂。考察了不同体积比的乙腈与1%三氯乙酸水溶液对SPE回收率的影响。分别取200.0 μL混合标准储备液Ⅰ与混合标准储备液Ⅱ于阴性样品中,采用不同体积比的乙腈与1%三氯乙酸水溶液提取,按1.3节方法处理后,在1.4.2节条件下进行HPLC分析。结果表明,乙腈与1%三氯乙酸水溶液体积比为4:1时(即80%乙腈水溶液(含0.2%三氯乙酸))作为提取溶剂时,各物质的回收率最好。

#### 2.1.6 SPE淋洗溶液的优化

淋洗溶液的选择对SPE前处理有着重要的影响,合适的淋洗溶液,既不会把吸附在SPE柱填料上面的目标产物洗掉,又能清洗掉一些干扰杂质。实验主要考察了甲醇、含1%甲酸的甲醇、乙腈、含1%甲酸的乙腈对14种药物回收率的影响。分别取200.0 μL混合标准储备液Ⅰ与混合标准储备液Ⅱ于阴性样品中,按1.3节方法处理,分别用甲醇、含1%甲酸的甲醇、乙腈、含1%甲酸的乙腈淋洗,在1.4.2节条件下进行HPLC分析。结果表明,乙腈与1%甲酸乙腈作为淋洗溶液时,14种化合物的回收率较低。1%甲酸甲醇作为淋洗溶液时,沙星类物质的回收率较低。甲醇作为SPE淋洗溶液时,各物质的回收率均较好。因此,选择甲醇为淋洗溶液。

#### 2.1.7 SPE洗脱溶液的优化

洗脱剂的选择和用量是前处理的关键,选择最佳洗脱剂需要兼顾14种化合物的回收率以及洗脱剂的用量,实验考察了含0.5%、1%、2%、3%、4%、5%氨水的甲醇对目标物的洗脱效果。分别取200.0 μL混合标准储备液Ⅰ与混合标准储备液Ⅱ于阴性样品中,按1.3节方法处理,依次用不同含量的氨水甲醇洗脱,在1.4.2节条件下进行HPLC分析。结果表明,当洗脱剂中氨水含量为2%时,各化合物的回收率均达到79%以上,考虑到碱性太强可能会对目标物的IMS响应有抑制作用,因此选择2%氨水甲醇作为洗脱溶剂。同时考察了2%氨水甲醇的洗脱体积,结果发现1.0 mL的2%氨水甲醇即可将各化合物完全洗脱。因此,洗脱溶液体积取1.0 mL。

### 2.2 方法学考察

#### 2.2.1 专属性和迁移时间

溶剂空白测定如图3所示,在各物质出峰的位置(均在11 min之后)无干扰峰出现,表明方法专属性良好。

**图3:**
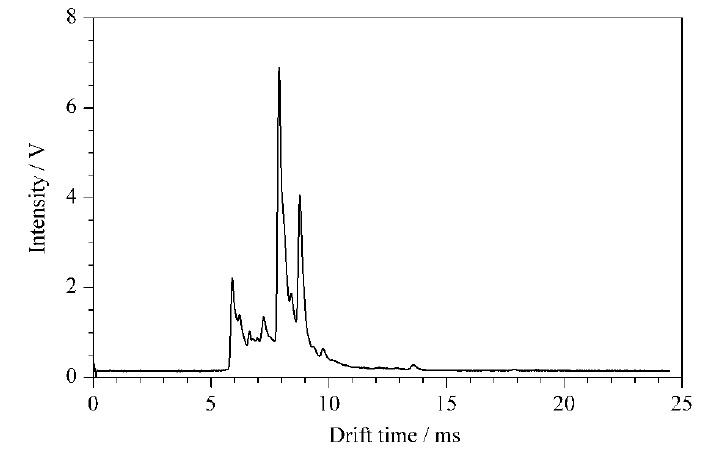
溶剂空白的离子迁移谱图

为了消除温度、气压等外界因素的影响,通常采用迁移时间和约化迁移率(*K*_0_)对各物质进行定性^[13]^。

*K*_0_= $\frac{L^{2}}{t_{d}U}$ × $\frac{P}{29.92}$ × $\frac{273.15}{T}$

其中*L*为迁移管的长度;*t*_d_为迁移时间;*U*为迁移管电压;*P*为环境气压;*T*为迁移管温度。根据公式可以得出*K*_0_·*t_d_*为定值,当仪器参数一定时,就可以用校正物质的*K*_0_来计算被测物质的*K*_0_。

在上述优化条件下,仪器校正后,取各IMS标准溶液依次注入IMS仪,在1.4.1节条件下进行分析,各物质的迁移时间和*K*_0_见表2,各物质(10 mg/L)的IMS谱图见图4。

**表2:** ESI-IMS法测定14种抗菌药物的迁移时间、约化迁移率及检出限(*n*=6)

No.	Compound	Migration time/ms	K_0_/(cm^2^/(V·s))	LOD/(mg/L)	MLOD^*^/(μg/g)
1	fluconazole	11.85±0.02	1.242±0.002	0.25	1.0
2	norfloxacin	12.48±0.03	1.179±0.002	0.25	1.0
3	ciprofloxacin	12.74±0.02	1.155±0.002	0.25	1.0
4	pefloxacin	12.83±0.02	1.147±0.002	0.25	1.0
5	bifonazole	13.27±0.02	1.108±0.002	0.05	0.2
6	sarafloxacin hydrochloride	13.71±0.03	1.073±0.002	0.25	1.0
7	miconazole nitrate	14.15±0.02	1.040±0.002	0.10	0.4
8	clotrimazole	11.46±0.02	1.284±0.002	0.05	0.2
9	enoxacin	12.34±0.03	1.192±0.003	0.30	1.2
10	naftifine hydrochloride	12.65±0.02	1.163±0.002	0.05	0.2
11	econazole nitrate	13.69±0.02	1.075±0.002	0.05	0.2
12	ofloxacin	13.86±0.02	1.061±0.001	0.25	1.0
13	difloxacin hydrochloride	14.12±0.02	1.042±0.002	0.20	0.8
14	ketoconazole	16.39±0.02	0.898±0.001	0.25	1.0

*MLOD(μg/g)=(LOD(mg/L)×1.0(mL))/0.25 g.

**图4:**
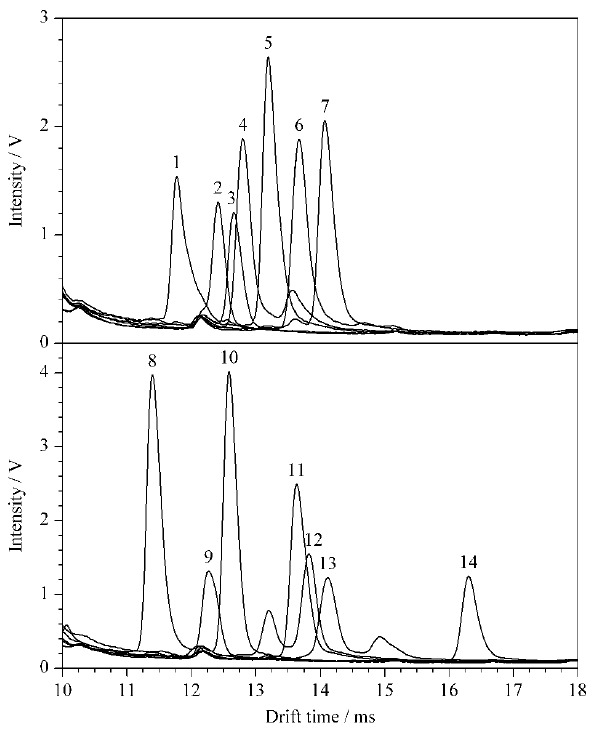
14种抗菌药物的离子迁移谱图

#### 2.2.2 IMS检出限

将各物质单标准储备溶液用80%乙腈水溶液(含0.2%三氯乙酸)稀释至一定浓度,加入阴性样品中,按1.3节方法处理,在1.4.1节条件下进行IMS分析,以*S/N*为3时的浓度为各物质的检出限,结果见表2。

#### 2.2.3 迁移时间测定重复性的考察

将各物质的IMS标准溶液每个月内随机选5天进行IMS分析,在1.4.1节条件下平行采集6次,观察3个月内各物质迁移时间的稳定性。14种化合物迁移时间的标准偏差均在0.05 ms以内,且迁移时间的RSD均在0.5%以内,表明迁移时间的重复性较好。

#### 2.2.4 SPE回收率考察

取已知阴性的乳剂化妆品样品18份,每份约0.25 g,置于15 mL离心管中,分别加入100.0、150.0、200.0 μL混合标准储备液Ⅰ(每个水平3份)与混合标准储备液Ⅱ(每个水平3份)。按1.3节方法处理,在1.4.2节条件下进行HPLC分析,结果见表3。

**表3:** 液相色谱法测定14种抗菌药物在阴性样品中3个水平下的加标回收率和精密度(*n*=3)

No.	Compound	Added/ (μg/g)	Found/ (μg/g)	Recovery/ %	RSD/ %
1	fluconazole	39.92	35.90	89.9	1.2
		59.88	51.73	86.4	1.0
		79.84	72.94	91.4	1.0
2	norfloxacin	39.84	37.61	94.4	1.4
		59.76	54.61	91.4	2.1
		79.68	71.50	89.7	1.6
3	ciprofloxacin	33.24	29.18	87.8	2.1
		49.86	44.62	89.5	6.1
		66.48	61.28	92.2	3.8
4	pefloxacin	28.52	23.64	82.9	0.9
		42.78	36.78	86.0	0.6
		57.04	46.54	81.6	0.7
5	bifonazole	39.44	34.66	87.9	1.4
		59.16	51.95	87.8	1.3
		78.88	68.17	86.4	1.7
6	sarafloxacin	34.32	26.81	78.1	0.1
	hydrochloride	51.48	40.76	79.2	0.2
		68.64	55.91	81.5	0.2
7	miconazole nitrate	39.84	32.22	80.9	1.5
		59.76	49.17	82.3	1.1
		79.68	66.77	83.8	4.9
8	clotrimazole	39.80	34.18	85.9	0.2
		59.70	53.90	90.3	0.1
		79.60	64.61	81.2	0.1
9	enoxacin	36.60	32.34	88.4	0.6
		54.90	48.06	87.5	0.6
		73.20	59.27	81.0	0.4
10	naftifine	40.00	34.15	85.4	0.1
	hydrochloride	60.00	51.92	86.5	0.7
		80.00	64.69	80.9	0.1
11	econazole nitrate	39.88	34.05	85.4	0.1
		59.82	51.26	85.7	0.8
		79.76	64.50	80.9	0.1
12	ofloxacin	39.88	35.02	87.8	0.1
		59.82	51.98	86.9	0.3
		79.76	67.85	85.1	0.4
13	difloxacin	38.84	34.47	88.7	0.6
	hydrochloride	58.26	52.12	89.5	1.0
		77.68	66.66	85.8	0.1
14	ketoconazole	39.92	33.91	84.9	0.5
		59.88	53.20	88.9	0.3
		79.84	74.51	93.3	0.2

### 2.3 实际样品的检测

对25批祛痘类化妆品进行测定,按1.3节方法处理后,进行IMS分析。结果有1批化妆品检测到与硝酸益康唑迁移时间一致的迁移峰,其IMS谱图见图5a;而未经SPE处理的样品的IMS谱图见图5b,在12.45 ms处存在与诺氟沙星、12.83 ms处存在与培氟沙星等物质相近的峰,易造成假阳性结果。对比SPE前后的IMS谱图,可以看出经SPE处理后峰高可达到2 V以上,未经SPE处理的峰高仅为0.05 V左右,说明样品得到了净化和富集,降低了基质抑制效应,避免了假阴性结果。同时采用本文建立的HPLC法检测上述祛痘类化妆品,与IMS检测结果一致,阳性样品的液相色谱图见图6,HPLC方法测定出硝酸益康唑的添加量为2.3 μg/g,与文献报道化妆品中抗菌药的非法添加量^[21]^(氧氟沙星添加量高至36517.1 μg/g,灰黄霉素添加量最低为1.0 μg/g, 无硝酸益康唑的非法添加量)相比,硝酸益康唑的添加量较低,如不经SPE处理,有可能造成假阴性结果,进一步说明SPE处理的必要性。

**图5:**
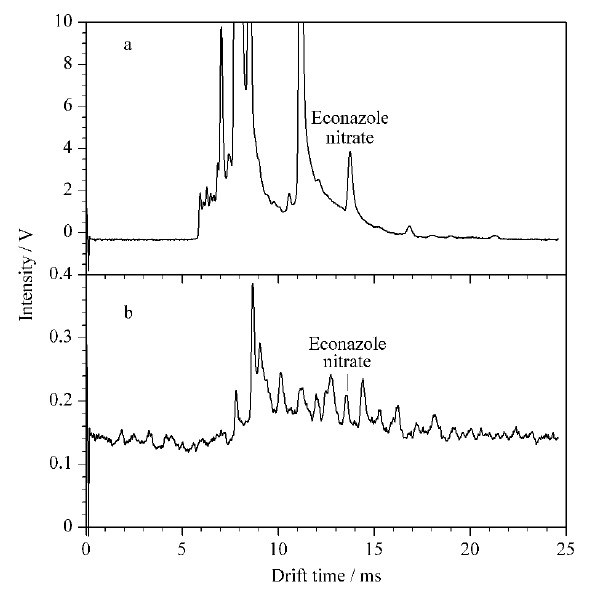
经(a)MCX固相萃取和(b)直接提取的阳性样品的离子迁移谱图

**图6:**
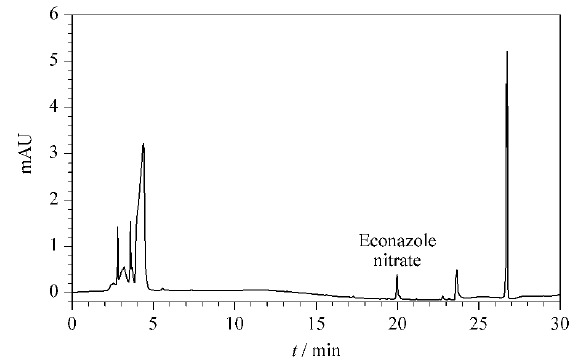
阳性样品的HPLC图

## 3 结论

本实验以14种抗菌药物为例,选择并优化了SPE前处理方法,建立了祛痘类化妆品中非法添加化学药物的快速筛查方法,在后续实际工作中,可进一步扩大非法添加谱库种类。该方法相较于直接提取法,可以消除大部分表面活性剂、基质增稠剂等干扰杂质,大幅降低基质对IMS的抑制效应,显著减少IMS仪器清洗和维护的频次,并对提取液进行了浓缩,提高了灵敏度,进而有效降低了假阳性和假阴性的发生。虽然SPE前处理增加了分析时间,但平均增加分析时间有限,且可放在快检车中进行现场操作。净化后的样品溶液可直接采用本文建立的HPLC方法验证准确性,大大提升了工作效率。该方法快速、高效、灵敏度高,为化妆品中非法添加筛查检测提供了一种有效的分析手段,同时也扩大了IMS的应用范围,拓宽了快检研究思路。
